# Acarbose-Induced Pneumatosis Cystoides Intestinalis

**DOI:** 10.1210/jcemcr/luaf199

**Published:** 2025-09-24

**Authors:** Ying Yuan, Xun Hou, Xiaopei Cao, Meilin Ding, Lei Su

**Affiliations:** Department of Geriatrics, First Affiliated Hospital of Sun Yat-sen University, Guangzhou, Guangdong 510080, China; Department of Gastrointestinal Surgery, First Affiliated Hospital of Sun Yat-sen University, Guangzhou, Guangdong 510080, China; Department of Endocrinology, First Affiliated Hospital of Sun Yat-sen University, Guangzhou, Guangdong 510080, China; Department of Geriatrics, First Affiliated Hospital of Sun Yat-sen University, Guangzhou, Guangdong 510080, China; Department of Geriatrics, First Affiliated Hospital of Sun Yat-sen University, Guangzhou, Guangdong 510080, China

**Keywords:** pneumatosis cystoides intestinalis, α-glucosidase inhibitor, side effect

## Abstract

This report details a case of acarbose-induced pneumatosis cystoides intestinalis (PCI) in a 47-year-old man with type 2 diabetes mellitus. The patient presented with a 1-month history of abdominal pain, bloating, and intermittent diarrhea, symptoms that developed after initiating acarbose, an α-glucosidase inhibitor, for diabetes management. Abdominal computed tomography (CT) revealed characteristic features of PCI, including gas-filled cysts within the right colonic wall and free intraperitoneal gas. Notably, laboratory investigations showed no signs of infection, and other findings were unremarkable. Following the discontinuation of acarbose, initiation of a fasting regimen, and short-term metronidazole, the patient's symptoms significantly improved. His blood glucose levels were subsequently managed with repaglinide. A follow-up CT scan 2 months later confirmed the complete resolution of intramural air. This case highlights PCI as a rare but important complication associated with α-glucosidase inhibitors. Prompt recognition, withdrawal of the causative agent, and conservative management are crucial for achieving favorable outcomes. This report further underscores the critical need for increased awareness of PCI in patients receiving α-glucosidase inhibitors and emphasizes the broader importance of pharmacovigilance in identifying rare adverse drug reactions. Further research is warranted to elucidate the precise causal relationship and to explore potential preventive strategies.

## Introduction

Acarbose, an α-glucosidase inhibitor, is a widely prescribed medication for managing type 2 diabetes mellitus. Its mechanism involves delaying carbohydrate absorption and mitigating postprandial hyperglycemia. While generally well tolerated, acarbose is known to cause gastrointestinal side effects. However, a less common but potentially serious complication linked to its use is pneumatosis cystoides intestinalis (PCI). PCI is characterized by gas-filled cysts within the submucosa or subserosa of the intestinal wall. Although often benign, PCI can lead to severe complications such as bowel perforation, volvulus, or obstruction, necessitating prompt recognition and management.

The association between acarbose and PCI is particularly intriguing and clinically significant. It highlights the importance of considering drug-induced etiologies, specifically those related to antihyperglycemic agents, when diagnosing PCI. Acarbose-induced PCI is thought to stem from the excessive fermentation of undigested carbohydrates in the colon, which leads to increased gas production that subsequently becomes trapped within the intestinal wall. This case report is crucial as it sheds light on a rare, and potentially underrecognized, adverse effect of a commonly prescribed medication. Furthermore, it underscores the need for increased awareness among clinicians to ensure timely diagnosis and management, including the discontinuation of the causative medication.

## Case Presentation

A 47-year-old man was admitted because of 1 month of abdominal pain and bloating. The pain was described as periumbilical colic, paroxysmal, and occasionally accompanied by diarrhea. His diarrhea improved after taking probiotics. He reported no vomiting or bloody stools. He also denied having fever or cough. His body temperature, heart rate, respiratory rate, and blood pressure were all within normal range. There was a decreased appetite. The patient lost 5 pounds (∼2.3 kg). He experienced diarrhea for about 2 weeks. The diarrhea did not improve after discontinuing metformin, but showed some relief after taking live combined Bifidobacterium and Lactobacillus tablets.

The patient's diabetes mellitus was first diagnosed 2 years ago, following a distal pancreatectomy for a pancreatic dermoid cyst (monodermal teratoma). All his islet autoantibodies (insulin autoantibodies, glutamic acid decarboxylase antibodies, islet cell cytoplasmic autoantibodies, zinc transporter 8 antibodies, insulinoma-associated-2 autoantibodies) were all negative (Table 1). He was initially prescribed oral voglibose 0.3 mg 3 times daily, and his fasting blood glucose levels were within the normal range. However, he discontinued the medication after 5 months and did not undergo subsequent regular glucose monitoring. One year later, he was admitted with symptoms of dry mouth and thirst. Laboratory results showed urine sugar (4+), urine ketone bodies (2+), a fasting glucose level of 313.2 mg/dL (reference range, 70.2-109.8 mg/dL [SI:3.9-6.1 mmol/L]), and a glycated hemoglobin A_1c_ (HbA_1c_) of 10.6% (reference range, 4.4%-6.4%). He was diagnosed with diabetic ketoacidosis, which resolved after receiving basal and bolus insulin. On discharge, his diabetes regimen included long-acting insulin glargine, metformin 1000 mg daily, and oral acarbose 50 mg 3 times daily. He discontinued metformin 2 weeks prior to the current admission, but his abdominal pain did not improve. His glucose levels remained stable until this admission for abdominal pain.

**Table 1.** Laboratory values

Laboratory markers	Values (SI units)	Reference range
WBC	5.93 ×10^9^/L	4-10 × 10^9^/L
Hemoglobin	143 g/L	130-175 g/L
Platelet	234 × 10^9^/L	100-300 × 10^9^/L
Hs-CRP	0.28 mg/L	0-10 mg/L
Procalcitonin	0.02 ng/mL	<0.05 ng/mL
Amylase, urine	67 U/L	32-641 U/L
ALT	11 U/L	1-40 U/L
AST	15 U/L	1-37 U/L
GGT	15 U/L	2-50 U/L
LDH	176 U/L	114-240 U/L
ALP	53 U/L	0-110 U/L
Albumin	39.3 g/L (591 µmol/L)	35-50 g/L (526.6-752.3 µmol/L)
Total bilirubin	11.5 µmol/L (0.67 mg/dL)	3-22 µmol/L (0.18-1.29 mg/dL)
Na+	145 mmol/L (145 mEq/L)	135-145 mmol/L (135-145 mEq/L)
K+	3.16 mmol/L (3.16 mEq/L)	3.5-5.3 mmol/L (3.5-5.3 mEq/L)
Cl-	106 mmol/L (106 mEq/L)	96-110 mmol/L (96-110 mEq/L)
Creatinine	69 µmol/L (0.78 mg/dL)	53-115 µmol/L (0.60-1.30 mg/dL)
HbA_1c_	6.18%	4.4%-6.4%
FBG	5.9 mmol/L (106.2 mg/dL)	2.9-6.0 mmol/L (52.2-108 mg/dL)
Fasting C-peptide	0.437 nmol/L (1.32 ng/mL)	0.4-1.7 nmol/L (1.21-5.14 ng/mL)
TC	4.08 mmol/L (157.8 mg/dL)	3.1-5.7 mmol/L (119.9-220.5 mg/dL)
TGs	1.19 mmol/L (105.4 g/dL)	0.33-1.7 mmol/L (29.2-150.6 g/dL)
HDL-c	1.02 mmol/L (39.44 mg/dL)	1.09-1.63 mmol/L (42.1-63.0 mg/dL)
LDL-c	2.44 mmol/L (95.35 mg/dL)	0-3 mmol/L (0-117.2 mg/dL)
IAA	0.02 COI	<0.9 COI
GAD	1.1 IU/mL	<10 IU/mL
ICA	0.06 COI	<0.9 COI
IA-2	<0.7 IU/mL	<10 IU/mL
ZnT8	<1.00 AU/mL	<10 AU/mL

Abbreviations: ALP, alkaline phosphatase; ALT, alanine transaminase; AST, aspartate transaminase; AU, Arbitrary Unit (ZnT8A was measured by a two-step indirect immunoassay, REF:C89051G,YHLO Biotech, using chemiluminescence technology, iFlash Immunoassay System); COI, cutoff index; FBG, fasting blood glucose; GAD, glutamic acid decarboxylase antibodies; GGT, γ-glutamyl transferase; HbA_1c_, glycated hemoglobin A_1c_; HDL-c, high-density lipoprotein cholesterol; Hs-CRP, high-sensitivity C-reactive protein; IA-2, insulinoma-associated-2 autoantibodies; IAA, insulin autoantibodies; ICA, islet cell cytoplasmic autoantibodies; LDH, lactate dehydrogenase; LDL-c, low-density lipoprotein cholesterol; TC, total cholesterol; TGs, triglycerides; WBC, white blood cells; ZnT8, zinc transporter 8 antibodies.

## Diagnostic Assessment

**Figure 1.**
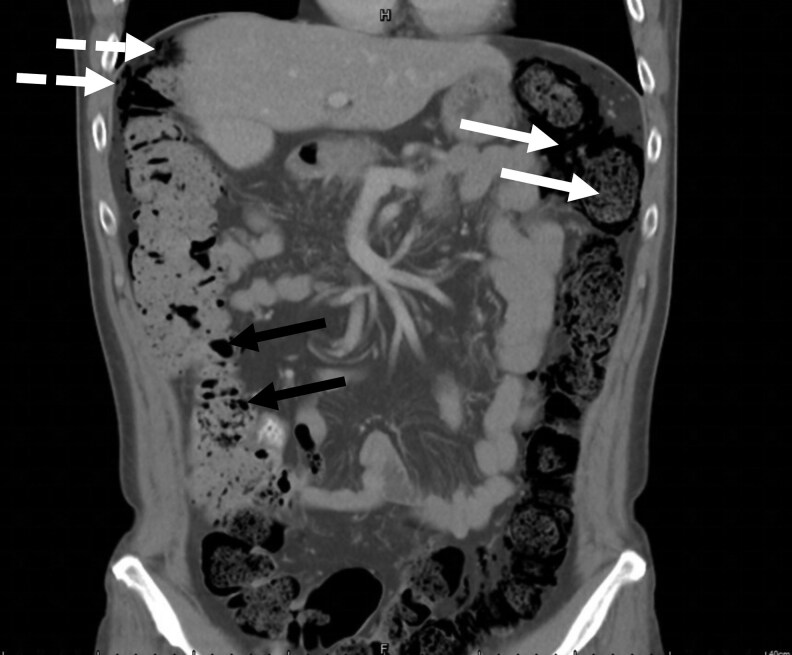
Abdominal computed tomography scans illustrate the course of pneumatosis cystoides intestinalis (PCI) in a patient with type 2 diabetes mellitus treated with acarbose. The coronal view demonstrates characteristic findings of PCI, including a honeycomb-like cluster of air-filled cysts (black arrows) within the right colonic wall, along with notable accumulation of fecal material and gas throughout the colon (white arrows), and scattered free intraperitoneal gas (dashed arrows).

On physical examination, the abdomen was soft, and there were no elevated infection markers (Table 1). Computed tomography (CT) imaging of the abdomen revealed a distinctive honeycomb-like cluster of air-filled sacs within the right colonic wall, consistent with PCI. Additionally, scattered free gas was observed in the abdominal cavity, and multiple areas of fecal and gas accumulation were noted in the colon (Fig. 1, coronal image, arrow).

## Treatment

Following the diagnosis, acarbose was discontinued. We initiating fasting and started a 7-day course of metronidazole. This multifaceted approach led to a significant improvement in the patient's symptoms. The patient gradually resumed a normal diet after 5 days.

## Outcome and Follow-up

**Figure 2.**
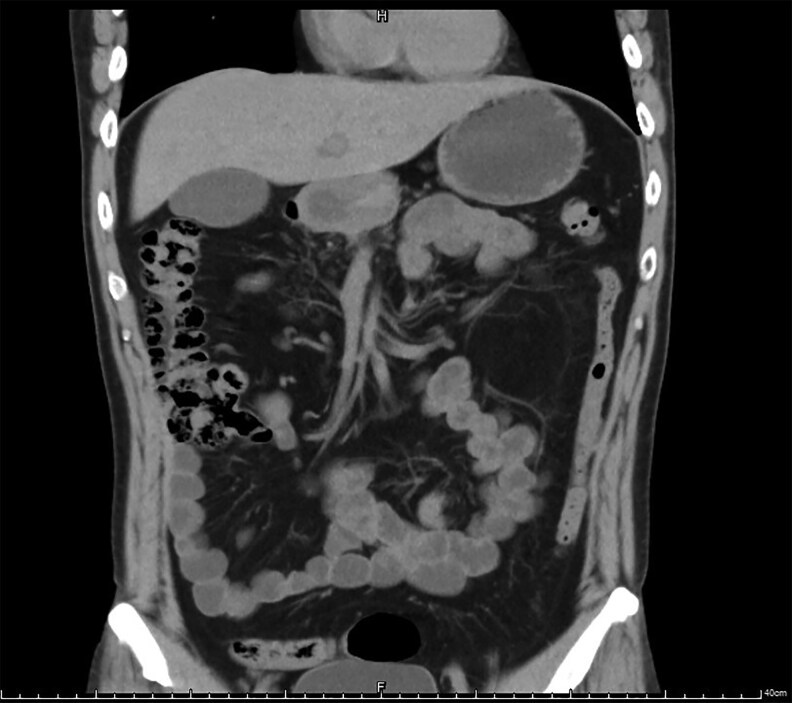
Abdominal computed tomography scans (coronal view), obtained 2 months after initial presentation and intervention, show complete resolution both of intramural air and free abdominal air.

For ongoing blood glucose management, the patient was prescribed repaglinide 0.5 mg 3 times daily. At discharge, his fasting blood glucose levels ranged from 5 to 7 mmol/L, and postprandial levels were between 144 and 252 mg/dL (reference range, 73.8-140.4 mg/dL [SI:4.1-7.8 mmol/L]). The patient reported no significant abdominal distension, pain, or other discomfort. A follow-up CT scan conducted 2 months later confirmed the complete resolution of both intramural air in the colon and free abdominal air (Fig. 2, coronal image).

## Discussion

This case highlights PCI as a rare complication of α-glucosidase inhibitors, emphasizing the importance of early recognition, discontinuation of the offending agent, and conservative management for favorable outcomes.

Previous case reports describe the occurrence of PCI in association with the use of α-glucosidase inhibitors [1-7]. We searched PubMed using “*alpha-glucosidase inhibitor*” and “*pneumatosis cystoides intestinalis*” as key words and selected articles in English, Chinese, and the Japanese language. A total of 28 cases were identified. Analysis of these cases revealed a typical presentation in men (56%) with a median age of 64 years (40-87 years). No sex predominance was observed. PCI often develops after prolonged use (frequently >1 year) of acarbose (50%) or voglibose (46.4%), particularly in older diabetic patients with comorbidities such as hypertension or autoimmune diseases (eg, systemic lupus erythematosus). While symptoms commonly include abdominal distention (50%), pain (42.9%), diarrhea (17.9%), or hematochezia (14.3%), 21.4% of cases remain asymptomatic. Conservative management (drug discontinuation + support care) led to resolution in 92.9% of cases, typically within weeks, while surgery was required in a few severe cases. Key risk factors identified include prolonged drug duration, high doses, and concomitant steroid or immunosuppressant use. Despite its low overall incidence, this association holds significant clinical relevance, illustrating the intricate interplay between pharmacological mechanisms and gastrointestinal pathophysiology. It also raises important question regarding routine monitoring and patient education concerning this potential complication. Further clinical studies are essential to clarify the causal relationship between α-glucosidase inhibitors and PCI. Additionally, future research should delve into the pathophysiological mechanisms of PCI, particularly examining the effect of these medications on gut microbiota, gas metabolism, and changes in intestinal permeability. Developing screening and prevention strategies targeting high-risk patients is also vital to reduce the incidence of PCI.

By presenting this case, we aim to contribute to the growing body of evidence on acarbose-induced PCI, reinforce its recognition as a differential diagnosis in patients presenting with abdominal symptoms, and encourage further research into its risk factors and preventive strategies. This case also serves as a timely reminder of the critical importance of pharmacovigilance in identifying and managing rare adverse drug reactions, particularly in individuals with diabetes.

## Learning Points

- Persistent abdominal pain in patients treated with α-glucosidase inhibitors for a sustained period of time should raise suspicion for pneumatosis cystoides intestinalis. A detailed history regarding acarbose use is crucial in diabetic patients.
- A detailed history of medication usage (including α-glucosidase inhibitor use) should be obtained in patients older than 45 with diabetes.
- Acarbose-associated pneumatosis cystoides intestinalis can be diagnosed via abdominal CT, and most cases revolve with conservative treatment.

## Contributors

All authors made individual contributions to the authorship. Y.Y., X.H., X.C., M.D., and L.S. were involved in the patient's diagnosis and management. Y.Y. and L.S. wrote and submitted the manuscript. All authors reviewed and approved the final draft.

## Data Availability

Original data generated and analyzed during this study are included in this published article. Complete references available on request.

## Abbreviations

CT: computed tomography
HbA_1c_: glycated hemoglobin A_1c_
PCI: pneumatosis cystoides intestinalis
